# WECAREADVISOR: CAREGIVER USE OF A DIGITAL PLATFORM TO MANAGE DEMENTIA-RELATED BEHAVIORAL SYMPTOMS

**DOI:** 10.1093/geroni/igae098.2039

**Published:** 2024-12-31

**Authors:** Laura Gitlin, Vincent Davis, Nicole Bouranis, Leslie McClure, Helen Kales, Melinda Webster

**Affiliations:** Drexel University, Philadelphia, Pennsylvania, United States; University of California Davis, Davis, California, United States; Yale University School of Medicine, New Haven, Connecticut, United States; Saint Louis University, St. Louis, Missouri, United States; University of California Davis, Davis, California, United States; Drexel University, Philadelphia, Pennsylvania, United States

## Abstract

Dementia-related behavioral and psychological symptoms are undertreated and have negative consequences for people with dementia and their caregivers. Families typically do not have access to dementia education and effective management strategies. WeCareAdvisor is an online platform providing caregivers with dementia information, daily tips, and a 4-step systematic approach for describing behaviors, investigating underlying causes, creating a list of tailored strategies (prescriptions), and evaluating their effectiveness (DICE). It is being tested in a randomized trial to evaluate effects on caregiver wellbeing and behavioral symptoms. This paper reports utilization patterns of 240 caregivers under two “prompting” conditions to use the platform: high-intensity (telephone plus email reminders) and low-intensity (email reminders only). A total of 999 DICE sessions were started of which 347 (35%) sessions were evaluated. Caregivers sought strategies for all 13 behaviors included in the platform ranging from 141 DICE sessions for Anxiety (14%) to 31 sessions for Hallucinations (3%). Caregivers in the high-intensity prompt group (n=46) had more DICE sessions (296, mean=6.4, SD=4.5) compared to 163 sessions in the low-intensity prompt group (n=41, mean=4.0, SD=3.5), generated 82% more prescriptions, and requested close to 3 times more additional strategies. Of 171 study completers, an average of 4.5 (SD=3.8) DICE sessions were conducted over 6-months (range 1-19). Caregivers engaged with WeCareAdvisor to obtain strategies for managing common behavioral symptoms; however, only 1/3 evaluated their effectiveness. Caregivers used the online platform more when prompted by telephone/email than by email alone. This utilization pattern has significant implications for cost and scaling of the WeCareAdvisor.

